# Quantitative Evaluation of DNA Methylation Patterns for *ALVE* and *TVB* Genes in a Neoplastic Disease Susceptible and Resistant Chicken Model

**DOI:** 10.1371/journal.pone.0001731

**Published:** 2008-03-05

**Authors:** Ying Yu, Huanmin Zhang, Fei Tian, Larry Bacon, Yuan Zhang, Wensheng Zhang, Jiuzhou Song

**Affiliations:** 1 Department of Animal & Avian Sciences, University of Maryland, College Park, Maryland, United States of America; 2 United States Department of Agriculture (USDA), Agricultural Research Service (ARS), Avian Disease and Oncology Laboratory, East Lansing, Michigan, United States of America; 3 College of Animal Sciences, China Agricultural University, Haidian, Beijing, China; National Institute on Aging, United States of America

## Abstract

Chicken endogenous viruses, *ALVE* (Avian Leukosis Virus subgroup E), are inherited as LTR (long terminal repeat) retrotransposons, which are negatively correlated with disease resistance, and any changes in DNA methylation may contribute to the susceptibility to neoplastic disease. The relationship between *ALVE* methylation status and neoplastic disease in the chicken is undefined. White Leghorn inbred lines 7_2_ and 6_3_ at the ADOL have been respectively selected for resistance and susceptibility to tumors that are induced by avian viruses. In this study, the DNA methylation patterns of 3∼6 CpG sites of four conserved regions in *ALVE*, including one unique region in *ALVE1*, the promoter region in the *TVB* (tumor virus receptor of ALV subgroup B, D and E) locus, were analyzed in the two lines using pyrosequencing methods in four tissues, *i.e*., liver, spleen, blood and hypothalamus. A significant CpG hypermethylation level was seen in line 7_2_ in all four tissues, *e.g*., 91.86±1.63% for *ALVE* region2 in blood, whereas the same region was hemimethylated (46.16±2.56%) in line 6_3_. CpG methylation contents of the *ALVE* regions were significantly lower in line 6_3_ than in line 7_2_ in all tissues (*P*<0.01) except the *ALVE* region 3/4 in liver. RNA expressions of *ALVE* regions 2 and 3 (PPT-U3) were significantly higher in line 6_3_ than in line 7_2_ (*P*<0.01). The methylation levels of six recombinant congenic strains (RCSs) closely resembled to the background line 6_3_ in *ALVE*-region 2, which imply the methylation pattern of *ALVE*-region 2 may be a biomarker in resistant disease breeding. The methylation level of the promoter region in the *TVB* was significantly different in blood (*P*<0.05) and hypothalamus (*P*<0.0001), respectively. Our data disclosed a hypermethylation pattern of *ALVE* that may be relevant for resistance against ALV induced tumors in chickens.

## Introduction

Epigenetic information is heritable but not encoded in the DNA sequence [1]. DNA methylation is an important epigenetic mechanism for pretranscriptional control, and plays an essential role in regulating genes' expression and maintaining cellular function [2]. DNA methylation is restricted to CpG dinucleotides in mammals, which commonly locate on transposable elements and promoters [3]. Numerous studies have shown that changes in DNA methylation patterns may contribute to neoplastic disease or cancer susceptibility [4], [5], [6], [7], [8], [9], [10], [11]. The nonrandom occurrence and observed patterns of CpG-rich methylation events further suggest that gene-specific methylation provides potentially useful markers for molecular diagnostics and detection of neoplastic disease risks [12].

Neoplastic diseases are defined as any malignant growth or tumor resulted from abnormal or uncontrolled cell division. It may spread to other parts of the body through the lymphatic system or the blood stream. Neoplastic diseases are a serious concern to the poultry industry due to the cost of routine vaccination against an herpesvirus that induces Marek's disease lymphomas [13] and the eradication of avian leukosis viruses from breeders [14]. Avian leukosis viruses (ALVs) are associated with a variety of tumors including lymphoid leukosis, myeloblastosis, erythroblastosis, osteopetrosis, myxosarcomas and fibrosarcomas. The ALVs are retroviruses and have six subgroups [14]. Among the avian retroviruses, *ALVE* is the unique endogenous virus (*ev*). The other five ALVs, including ALVA, ALVB, ALVC, ALVD and ALVJ, are exogenous viruses [15], [16]. *ALVE* is inherited as LTR (long terminal repeat) retrotransposons [17], [18], [19], [20].

Chicken *ALVE* loci are present in the genome of most chickens and can be inherited as normal cellular genes [21]. Since *ALVE* were first identified in chicken cells as ALV group-specific antigens [22], more than 22 *ALVE* loci have been defined in the genome of White Leghorn chickens (Crittenden, 1991). Some *ALVE* are actively transcribed from their inherited chromosomal locations, whereas others (*e.g*., *ALVE1*) are silent [23]. *ALVEs* relevant to this study are *ALVE1*, *ALVE2* and *ALVE3*. *ALVE1* is located on chromosome 1, *ALVE2* is located on chromosome 2, and *ALVE3* is found on one of the many microchromosomes. Inbred line 6_3_ has one copy of *ALVE1* and *ALVE3* whereas line 7_2_ has one copy of *ALVE1* and *ALVE2* [24]. The cellular receptor of each ALV subgroup is encoded by a tumor virus locus (*TV*), which mediates viral entry. *TVA* and *TVC* encode the cellular receptors for ALV of subgroup A and C, respectively, *TVB* is the cellular receptor gene for ALV of subgroup B, D and E [25], [26].

The highly inbred line 6_3_ at the Avian Disease and Oncology Laboratory (ADOL) are resistant to Marek's disease (MD) tumors but susceptible to both Marek's disease virus (MDV) and avian leukosis viruses (ALV), whereas the highly inbred line 7_2_ chickens are resistant to ALV (subgroup A, B, D, and E) but susceptible to both MDV and MD tumors [27]. Therefore, these inbred lines constitute unique models for epigenetic research by making it possible to explore the mechanisms of resistance and susceptibility to neoplastic diseases [27], [28] .

In this research, we hypothesize that the methylation status of *ALVE* and *TVB* genes are associated with resistance and susceptibility to neoplastic disease. To examine the hypothesis, we did DNA methylation analysis by pyrosequencing, which was recently developed as a quantitative technique to detect changes in methylation patterns [29], [30]. This technique is advantageous for analyzing and quantifying the degree of methylation of multiple CpG sites in one reaction. In order to advance our understanding of genetic mechanisms and to develop a better strategy for disease prevention, we have investigated epigenetic differences in methylation patterns between lines 6_3_ and 7_2_, as well as six recombinant congenic strains (RCSs) that developed from background inbred line 6_3_ and donor line 7_2_. This unique model system provides a way to elucidate mechanisms that may induce susceptibility or enhance resistance to viral induced tumors.

We first analyzed variations of DNA methylation patterns in four consensus regions among *ALVE1*, *2* and *3*, including one region unique for *ALVE1* as well as the promoter region of the *TVB* gene in ALV-susceptible line 6_3 _and ALV-resistant line 7_2 _using pyrosequencing. Next, we investigated line-specific gene expression for *ALVEs* and *TVB* with real-time RT-PCR. Subsequently, we characterized potential differences in *ALVEs PPT* (polypurine tract) region and *U3* region of 3′*LTR* between the lines by DNA sequencing. The effects of *ALVEs* and *TVB* methylation status on the tumor susceptible and resistant lines and six recombinant congenic strains are discussed. Our results suggest that variation in methylation patterns of *ALVE, TVB* and variations in the *PPT* region may be factors that contribute to viral induced tumors in chicken.

## Results

### Profiling DNA methylation patterns of *ALVEs* in two inbred chicken lines

**Figure 1:**
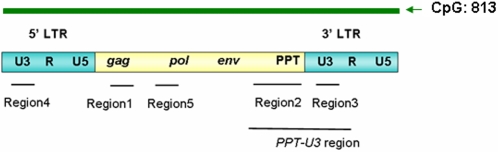
Four conserved regions (region1∼4) on *ALVEs* and one unique region (region5) of *ALVE1* for pyrosequencing and real-time RT-PCR. 5′LTR: 5′ long terminal repeat; U3: U3 region in LTR; R: R region in LTR; U5: U5 region in LTR; *gag*: *gag* gene; pol: *pol* gene; *env*: env gene; PPT: polypurine tract; 3′LTR: 3′ long terminal repeat of *ALVE*. *PPT-U3* region: combined *PPT* and *U3* regions for real-time RT-PCR. Green arrow shows the CpG islands that including 813 CpG sites within 7525 bp of *ALVE1* (Blat result from UCSC Genome Browser website).

**Figure 2:**
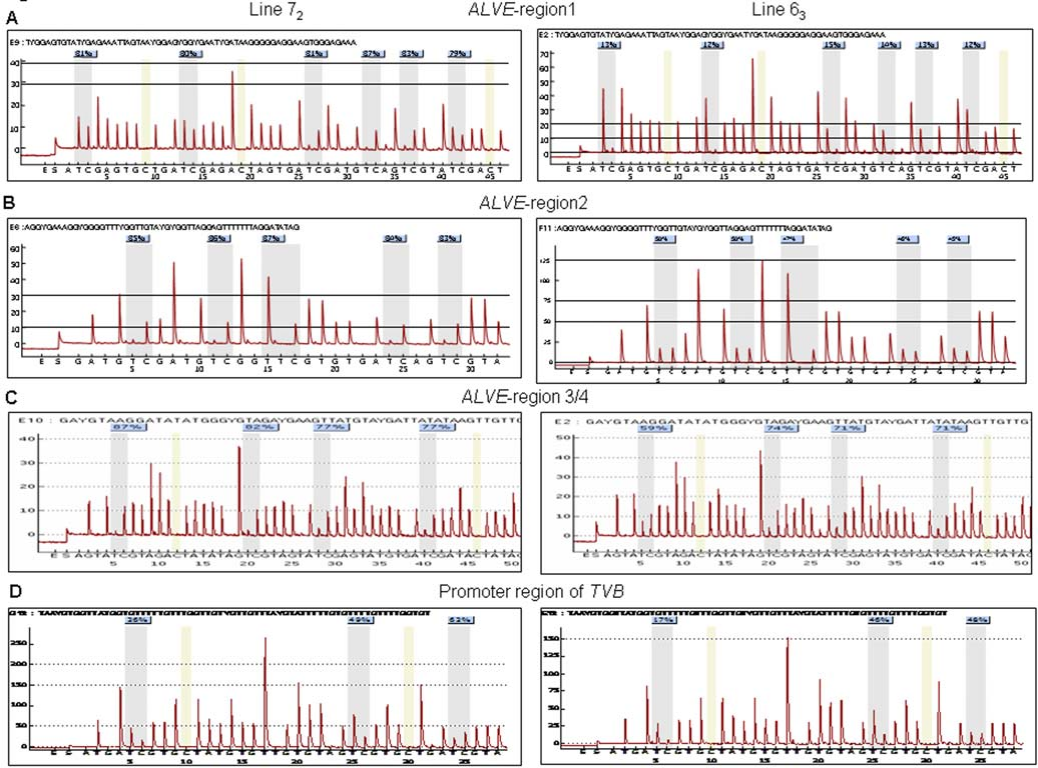
Representative pyrograms for each gene region in line 7_2_ and 6_3_. The percentage on each CpG site is the methylation percentage of ^m^C/(^m^C+C) on this site. ^m^C: methylated cytosine, C: unmethylated cytosine. The sequence above the peak is the sequence to assay. The x axes are the dispensation nucleotides to the sequencing reaction based on the assayed sequences. The y axes show light emission obtained as relative light units. A: *ALVE* region1 in blood (the mean value of methylation percentage is 81.8±2.9 in line 7_2_ and 13.2±1.2 in 6_3_); B: *ALVE* region2 in blood (the mean value of methylation percentage is 85±1.6 in line 7_2_ and 47.6±2.3 in 6_3_); C: *ALVE* region3/4 in blood (the mean value of methylation percentage is 80.8±4.8 in line 7_2_ and 68.8±6.7 in 6_3_); D: Promoter region of *TVB* in Hypothalamus (the mean value of methylation percentage is 45.7±14.8 in line 7_2_ and 37.0±14.1 in 6_3_).

The whole *ALVE1* is a CpG island that including 813 CpG sites within 7.5 kp based on the BLAT with UCSC Genomic Browser (Figure 1). To determine the DNA methylation levels of *ALVEs*, we selected four conserved regions among *ALVE1, 2* and *3* that cover the *gag* gene, *PPT* region and *U3* regions of 5′ and 3′ *LTR*, then quantitative evaluated the methylation differences between inbred chicken line 6_3_ and line 7_2_ using pyrosequencing method in four different tissues (blood, liver, spleen and hypothalamus) (Figure 1). Figure 2A, 2B and 2C show the typical methylation pyrograms for the six CpG sites in *ALVE* region 1 (*p19* matrix protein in *gag* gene), the five CpG sites in *ALVE* region 2 (Direction Repeat Sequence, DRS, adjacent to 3′ *LTR* of *ALVE*), and the four CpG sites in *ALVE* region 3/4 (*U3* region in 5′ and 3′ *LTR* of *ALVE*) in blood of line 7_2_ and line 6_3_, respectively. The averages from five individuals showed that hypermethylated CpG sites were identified in blood of line 7_2_ , 85.56±2.66% for *ALVE* region 1 and 91.86±1.63% for *ALVE* region 2, whereas the same regions are hypomethylated (10.05±2.56%) and hemimethylated (46.16±3.41%) in the line 6_3_ (Figure 2). The measurements using pyrosequencing technique for each conserved region of *ALVE* were proved to be highly reproducible between biological replicates. Moreover, the methylation percentages of region 2 in *ALVE* from bisulfite cloning and sequencing methods were very comparable to that from pyrosequencing as shown in Figure S2, which validated the pyrosequencing data.

**Figure 3:**
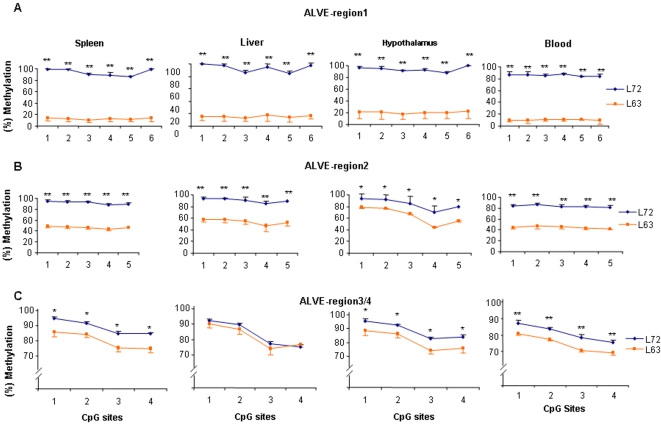
Results of quantitative DNA methylation analysis on conserved regions of *ALVEs* in spleen, liver, hypothalamus and blood. **P<0.0001. *P<0.01. A: Methylation levels for each CpG site in the *ALVE* region1. B: Methylation levels for each CpG site in the *ALVE* region2. C: Methylation levels for each CpG site in the *ALVE* region3 and 4. *n* = 5 for each line.

In order to compare the DNA methylation differences of the conserved *ALVE* regions between line 7_2_ and line 6_3_ in spleen, liver, hypothalamus and blood, point-wise comparison method was carried out at each CpG site. As depicted in Figure 3A and 3B and Table S1 and S2, we found that the methylation contents for each CpG site in *ALVE* region 1 and *ALVE* region 2 were very significantly higher in the four tissues in line 7_2_ than in line 6_3_ (*P*<0.0001), except for *ALVE* region 2 in hypothalamus (*P*<0.01). Hypermethylated patterns (methylation level >60%) in *ALVEs* region 1 and 2 existed in line 7_2_, while line 6_3_ showed a hypomethylated pattern (methylation level <30%) in *ALVEs* region 1 and a hemimethylated pattern (30%< methylation level <60%) in *ALVEs* region 2 except for a hypermethylated pattern in the hypothalamus. As for *ALVEs* region 3/4, although line 7_2_ and line 6_3 _had a hypermethylated status in the four tissues, the DNA methylation level was significantly higher in line 7_2_ than in line 6_3_ in spleen (*P*<0.01), hypothalamus (*P*<0.01) and blood (*P*<0.0001), but not in the liver (*P*>0.5) (Figure 3C and Table S3).

### mRNA expression level of *ALVEs*

**Figure 4:**
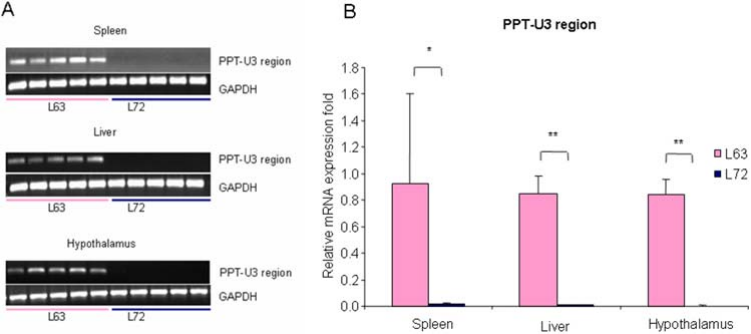
mRNA expression of *PPT- U3* region on 3′ *ALVE* in line 6_3_ and line 7_2_. A: RT-PCR results. B: Real-time quantitative RT-PCR analysis. *n* = 5 for each line. *P = 0.01; ** *P*<0.0001.

There is a typical retroviral polypurine tract (*PPT*) adjacent to the *U3* region of the 3′ *LTR* in *ALVEs* (Figure 1). The *PPT* is a short RNA sequence that generally serves as a primer for plus-strand DNA synthesis during reverse transcription and initiation of DNA synthesis, then, the *PPT* primer is accurately removed from nascent DNA to create a double-stranded, integration-competent DNA provirus. Due to the importance of *PPT-U3* region, real-time quantitative RT-PCR on the *PPT-U3* region was used to examine the transcriptional activation level of *ALVEs*. As shown in Figure 4A and 4B, RNA expression levels of the *PPT-U3* region were significantly higher in line 6_3_ than in line 7_2_ in the spleen (*P*<0.01), liver and hypothalamus (*P*<0.0001). There was no significant difference among tissues within either line (*P*>0.05).

### Variation of Polypurine Tract (*PPT*) Site among *ALVEs*

**Figure 5:**
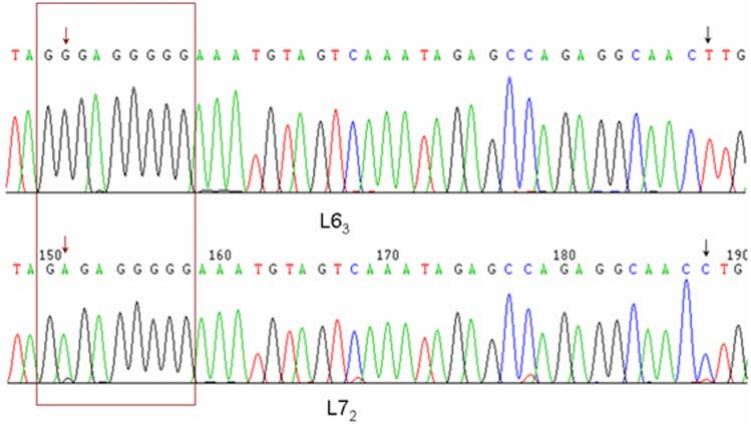
DNA sequencing of *PPT* region and partial *U3* region of 3′ *LTR* in *ALVE* between line 6_3_ and line 7_2_. Brown box shows the *PPT* region. Brown arrow shows one variation located on *PPT* region changed from G in line 6_3_ to A in line 7_2_. Black arrow shows another variation located in U3 region of 3′ *LTR* changed from T in line 6_3_ to C in line 7_2_. *n* = 11 for each line.

To further ascertain the genetic background of the two inbred lines and explore the potential mechanisms of methylation content influencing the mRNA expression levels, we did sequencing analysis for the *PPT-U3* region in *ALVE* as shown in Figure 5. We found two variation sites in the two lines. One variation located in the *PPT* site changed from guanine (GGGAGGGGG) in line 6_3_ to adenine (GAGAGGGGG) in line 7_2_, and another variation mutated from thymidine (T) in line 6_3_ to cytosine (C) in line 7_2_. Based on the distinct feature for *ALVE* in the two lines, it shows that the sequences of the *PPT* site for *ALVE1* and *ALVE3* are GGGAGGGGG (upper panel in Figure 5)_,_ and that of the *PPT* for *ALVE2* is GAGAGGGGG (lower panel in Figure 5).

### DNA methylation analysis of *ALVEs* region 2 in inbred lines and recombinant congenic strains

**Figure 6:**
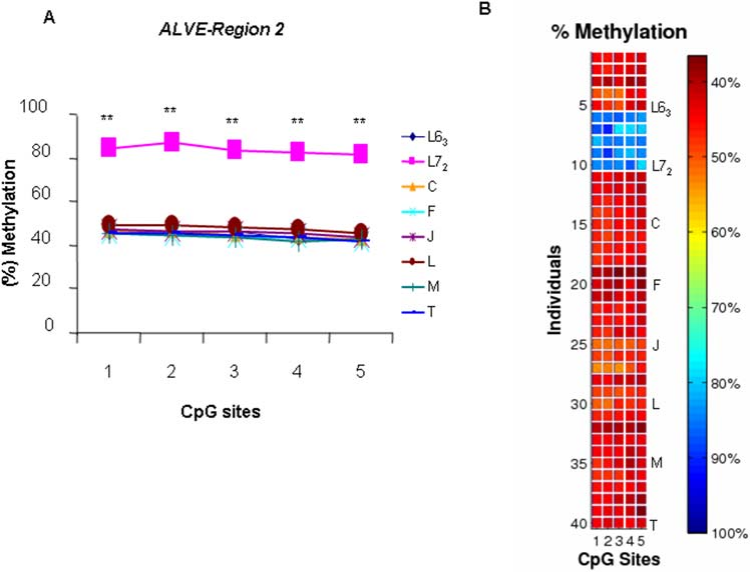
Methylation profiles of *ALVE*-region 2 in chicken inbred lines 6_3_ and 7_2_ and six recombinant congenic strains C, F, J, L, M and T. A. Statistical analysis results. ** *P*<0.0001. B. The methylation levels are displayed in the form of a matrix. The matrix contains the data obtained form all the samples. *n* = 5 for each line or RCS.

Due to the importance of *PPT* site in *ALVEs* region 2, the quantitative measurements of methylation level in the region 2 enable monitoring of epigenetic inheritance or non-inheritance of parental methylation patterns of *ALVEs* and help us to uncover the inner mechanisms between *ALVEs* and susceptibility or resistance of neoplastic diseases in the unique population. There are 19 recombinant congenic strains (RCSs) established by a cross between the two inbred line 7_2_ and line 6_3_. The F_1_ was then consecutively backcrossed to the background line 6_3_ twice. After more than 13 generations of sib-matings within strain, each RCS is expected to contain random 7/8 background line 6_3_ and 1/8 donor line 7_2_ genome. We measured the methylation status of the *ALVE*-region 2 in blood in the two parental lines and 6 randomly selected RCS C, F, J, L, M and T. We found that the methylation patterns can easily be categorized into two groups, as exemplified in Figure 6A and 6B. One of the categories is extremely similar within the six RCSs and is also similar to line 6_3_ than to line 7_2_ (*P*<0.0001). Specifically, the methylation patterns of six RCSs can be acknowledged and were inherited from the background line 6_3_, but not from the donor line 7_2_. The similarity of the methylation patterns of region 2 between RCSs and background line 6_3_ is mainly a result of the two backcrosses to line 6_3_, and it could be a potential biomarker to predict susceptibility of neoplastic disease in chickens.

### DNA methylation patterns of the *TVB* promoter region

**Figure 7:**
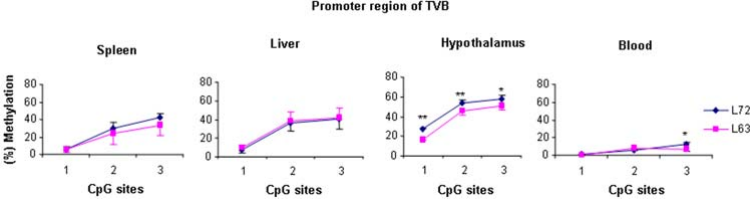
Results of quantitative DNA methylation analysis of inbred lines 6_3_ and 7_2_ in promoter region of *TVB* in spleen, liver, hypothalamus and blood. *n* = 5 for each line. **P<0.0001, * *P*<0.05.

**Figure 8:**
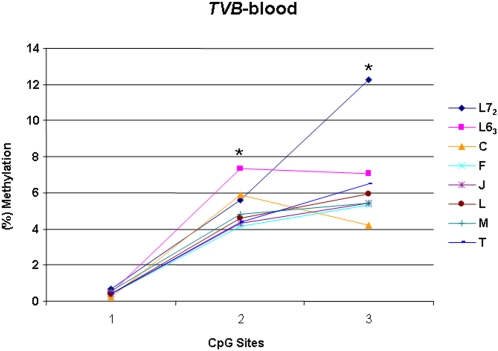
Results of quantitative DNA methylation analysis in promoter region of *TVB* in blood. Line 6_3_ and 7_2_ are two parental lines. C, F, J, L, M and T are six recombinant congenic strains (RCS) chicken. *n* = 5 for each line and RCS. *P<0.05.

The tumor virus B (*TVB*) locus is an important gene encoding the cellular receptor of avian leukosis virus, different *TVB* alleles can encode different receptors permitting infection by exogenous ALVB, or ALVD and endogenous ALVE. To uncover epigenetics factors contributing to susceptibility or resistance of neoplastic disease in chickens, we examined the DNA methylation status of the *TVB* gene. Figure 2D shows the presentative pyrograms for three CpG sites in the promoter region of the *TVB* gene in the hypothalamus tissue of both lines. The results show that the methylation level of *TVB* promoter was significantly higher in line 7_2_ than that in line 6_3_ (*P*<0.0001, Figure 7). Unlike the consensus methylation trends of the *ALVEs* regions 1 and 2 (Figure 3A and 3B), the promoter region of *TVB* shows tissue-specific methylation patterns (Figure 7) similar to the promoter region 3/4 in *ALVEs* (Figure 3C). As shown in Figure 7, CpG site 3 in the promoter region of *TVB* showed a hemimethylated pattern in the spleen, liver and hypothalamus in both lines, whereas a hypomethylated pattern was disclosed in the blood. In addition, CpG site 3 of *TVB* in blood was significantly higher in line 7_2 _than in line 6_3_ (*P*<0.05). We found some variations regarding methylation contents among the 6 RCSs, especially for CpG sites 2 and 3, although the methylation patterns of RCSs were similar to background line 6_3_ (Figure 8).

### mRNA expression level of *TVB*

**Figure 9:**
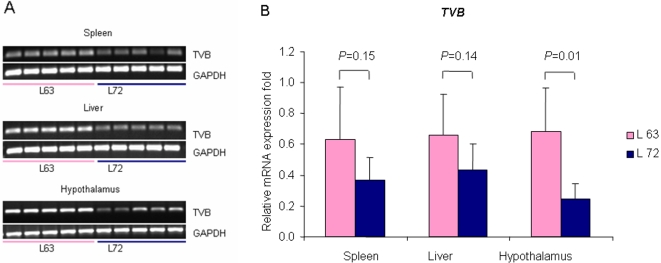
mRNA expression difference of *TVB* in line 6_3_ and line 7_2_. *P* values indicates the statistical significance for the differences of relative *TVB* mRNA expression levels between lines 6_3_ and 7_2_ in spleen, liver, and hypothalamus, respectively. A: RT-PCR results. B: Real-time quantitative RT-PCR analysis. *n* = 5 for each line.

The mRNA expression level of the *TVB* gene in different tissues between the line 6_3_ and line 7_2_ was further examined with real-time quantitative PCR. The results were shown in Figure 9A and 9B. We found that, in general, the mRNA expression levels of the *TVB* gene were higher in the liver, spleen and hypothalamus in line 6_3_ than in line 7_2_. However, this difference between the two lines was only statistically significant in the hypothalamus (*P*<0.05), differences were not significant in the spleen (*P* = 0.15) or the liver (*P* = 0.14) (Figure 9B).

### Association analysis between mRNA expression levels and DNA methylation contents

To further explore variation resources and clarify the association between mRNA expression and DNA methylation levels, two generalized linear models were used to quantitatively evaluate the effects of lines, tissues, and the methylation contents of *ALVEs* on the expression levels of *ALVE*s *PPT-U3* region in *ALVE* and the methylation contents of *TVB* on its expression levels. Statistical analysis revealed that the effect of chicken inbred lines on the mRNA expression levels of the *PPT-U3* region in 3′ *ALVE* was statistically significant (*P*<0.0001) (the first model). However, the effect of tissue on the mRNA expression of the *PPT-U3* region in *ALVE* was not statistically significant (*P*>0.05). A further analysis of the line-specific results indicated that the mRNA expression level of *PPT-U3* region in *ALVE* was significantly higher in line 6_3_ than in line 7_2_. Subsequently, we analyzed the association between mRNA expression level of the *PPT-U3* region in *ALVE* and DNA methylation level of the *ALVE* region 2 using the second model. It shows that the effect of DNA methylation level on the mRNA expression level of *PPT-U3* region in *ALVE* was statistically significant (*P*<0.0001).

**Figure 10:**
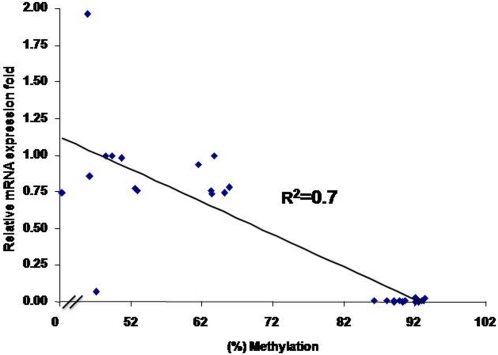
Regression analysis of mRNA expression level of *PPT-U3* region of *ALVE* and DNA methylation contents of *ALVE* region2.

Thereafter, we explored the relationship between DNA methylation level of *TVB* and mRNA expression level. There was a negative effect of methylation levels of the *TVB* promoter region on its mRNA expression from hypothalamus (*P*<0.05). Finally, a regression analysis was done for exploring the relationship between DNA methylation level of the region 2 in *ALVE* and RNA expression level of the *PPT-U3* region in *ALVE*. As shown in Figure 10, there was a higher negative relationship (R^2^ = 0.7) between them. Figure S3A and S3B also showed a negative relationship between mRNA expression level of *ALVE PPT-U3* region and methylation level of *ALVE*-region 1 (R^2^ = 0.7) or region 3/4 (R^2^ = 0.55). Taken together, we found that the higher the methylation levels, the lower the mRNA expression level for *PPT-U3* region of *ALVE* in all tissues (*P*<0.0001) and for the *TVB* gene in only the hypothalamus (*P*<0.05).

## Discussion

In this study, we observed distinct DNA methylation patterns of avian endogenous viruses (*ALVEs*) between the two inbred chicken lines, and also analyzed the DNA methylation pattern variations of *ALVE* and *TVB* genes. To the best of our knowledge, this is the first study in chickens to elucidate the variation of DNA methylation patterns variations in *ALVEs*.

**Figure 11:**
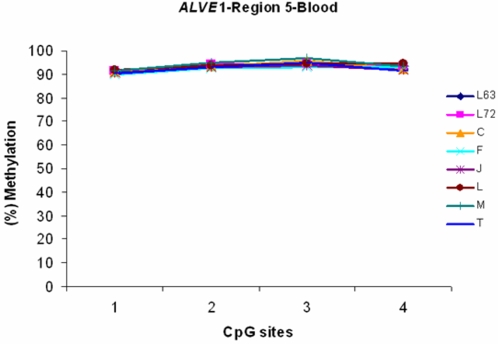
The results of DNA methylation analysis in a unique region of *ALVE*1 from blood in line 7_2_, line 6_3_ and RCS C, F, J, L, M and T blood. *n* = 5 for each line or strain. *P*>0.05.

The previous studies have reported that the line 6_3_ contains *ALVE1* and *ALVE3*, while line 7_2 _possesses *ALVE1* and *ALVE2* in the genome respectively [16], [31], [32], [33]. The reports were confirmed in our study as shown in Figure S1. Notably, we identified a unique region in *ALVE1* (named as region5, Figure 1) with four CpG sites and examined methylation status of the region in blood among the inbred line 6_3_ and 7_2_ and 6 RCSs as shown in Figure 11. We found that both inbred lines and six RCSs have nearly the same hypermethylation patterns. Therefore, our results not only provide further evidence and strong support for the previous finding that the complete *ALVE1* exists in both lines, but also indicate that *ALVE1* in the two lines may not contribute to the unique features of resistant and susceptibility of neoplastic diseases and possess inhibitory methylation [32], [33]. Since *ALVE1* did not have methylation differences between the both lines, any DNA methylation differences in conserved *ALVE* regions between line 7_2_ and line 6_3_ must be attributed to *ALVE2* and *ALVE3*, respectively.

*In vitro* and *in vivo* studies in mammals have shown that retrotransposons are generally hypermethylated in normal tissues, but are hypomethylated in malignant tissues [11], [34], [35], [36], [37], [38], [39]. *ALVE*s belong to LTR-retrotransposons in chicken genome. In this study, the methylation level of *ALVE* conserved regions in line 7_2_ was nearly twice to six times as high as that observed in line 6_3_, which is consistent with the hypermethylated retrotransposon frequently found in normal tissues of mammals [11], [40], [41]. The methylation variations detected in this study were large in size and probably represent a strong link between DNA hypermethylation pattern of *ALVE* and resistance to ALV in the chicken. Based on association analysis, an up-regulating effect of hypomethylation in *ALVEs* region 1 and 2 was observed on *ALVE PPT-U3* expression in line 6_3_, which was in contrast to a down-regulating effect of hypermethylation in line 7_2_. However, a directly transcriptional repression effect of *ALVEs* DNA methylation on gene expression level remains to be characterized.

Notably, the methylation pattern of *ALVE*-region 2, which possesses the primer *PPT*, could be an epigenetic biomarker and has an important potential value in prevention of chicken neoplastic disease. In our study, the similarity of the methylation pattern in *ALVE* region 2 between the RCSs and background line 6_3_ is mainly because of the two continuative backcrosses to line 6_3_. Based on sequencing analysis, we found that the 6 RCSs and background line 6_3_ have the exactly same sequences on this region without any alterations (Unpublished data). On the other hand, although the methylation patterns of 6 RCSs in this region are similar to their background line 6_3_, there are some variations in the methylation patterns among the 6 RCSs. Furthermore, we found some phenotypic variations in susceptible ALV infections among 6 RCSs (Private communication, unpublished data). The epigenetic profiles of this region in RCSs were likely to be transmitted from the line 6_3_, and they retained the parental methylation patterns after over 13 generations of sib-mating. These results suggest us to further build the association between the methylation patterns and the phenotypic variations, and explore the possibility of the region as a biomarker in resistant breeding.

The polypurine tract (*PPT*) of the long terminal repeat-retrotransposons and retroviruses is a short RNA sequence from which the second or plus-strand DNA synthesis is initiated [42]. The *PPT* sequence required for plus-strand initiation was the key step for the replication of retrotransposon and retroviruses [43]. Previous studies demonstrated that the mutation in both the 5′ and 3′ halves of the human immunodeficiency virus type 1 (HIV-1) *PPT* has an effect on virus replication and titer [44], [45]. One mutation discovered in the *PPT* of 3′ *ALVEs* in our study was involved a change from a guanine (GGG) in line 6_3_ to an adenine (GAG) in line 7_2_ (Figure 5). Further study *in vitro* is to examine the function of the mutation in the *PPT* of chicken *ALVE*s on neoplastic disease.

The *TVB* locus, a single copy located on chromosome 22, is very complex and has three alleles encoding different receptors to accommodate the viral entry of different subgroups [46]. The receptors encoded by two susceptible alleles TVB*S1 and TVB*S3 support viral entries of ALVB, ALVD and ALVE subgroups. TVB*R is the resistant allele and encodes no functional receptors. Previous researches found that the ALVE susceptibility between line 6_3_ and line 7_2_ should be dependent on allelic differences at the receptor *TVB* locus (line 6_3_ is susceptibility SNP *TVB*-S and line 7_2_ is resistant SNP *TVB-R*) [33], [47]. Our study showed that the methylation contents of *TVB* were moderately higher in line 7_2_ than in line 6_3_, and the mRNA expression levels were reversely lower in line 7_2_ than in line 6_3_. It is worth noting that we found a variation appeared coalescence with of resistance to ALVA and ALVB in the 6 RCSs, although they have the same susceptibility SNP *TVB*-S as their background line 6_3_ (Unpublished data). In this study, the methylation profiles of *TVB* promoter in six RCSs were much close to background line 6_3_ than to line 7_2_. However, some differences in the CpG site 2 and 3 among the six RCSs chickens were identified (Figure 8) and they may contribute to the varied ALVA or ALVB induced tumor incidences among the six RCSs in addition to the *TVA* genotype variations.

In conclusion, our data define the consensus CpG sites methylation patterns of the conserved *ALVE* regions in the ALV-resistant line 7_2_ and -susceptible line 6_3_ are attributed to *ALVE2* and *ALVE3*, respectively. The results disclose that the mRNA expression levels of *PPT-U3* in *ALVEs* and *TVB* gene are negatively associated with the CpG methylation status in the primer region of *ALVEs* and the promoter region of *TVB*, which suggest that the hypermethylaion profiles may contribute to ALV resistance in the chickens. The *ALVE*-region2, the *PPT* located region, could be considered as an epigenetic biomarker for resistant breeding against neoplastic disease in chicken.

## Materials and Methods

### Animal Samples

All samples were collected from highly inbred chickens of lines 6_3_, 7_2_, and recombinant congenic strains (RCS). Five 15 month old females from chicken line 6_3_ and line 7_2_, and five 12 month old females from line 6_3_, 7_2_ and six RCSs (C, F, J, L M and T) were tested. Heparinized blood was collected from each chicken prior to euthanasia. Then tissue samples from 15 month-olds chickens were obtained from three organs: hypothalamus, liver and spleen. Tissues were frozen in liquid nitrogen prior to storage at −80°C.

### DNA extraction and bisulfite treatment

DNA was extracted from 20 µl blood or 3 mm^3^ tissue samples using a phenol-chloroform method. DNA concentration was measured by a spectrophotometer (Bio-Rad). 1 µg DNA of each sample was treated with bisulfite with EZ DNA Methylation Golden Kit (ZYMO Research) as the manufacture's protocols. Bisulfite converted DNA was eluted in 20 µl elution buffer (ZYMO Research).

### PCR and pyrosequencing primers

PCR and pyrosequencing primers were designed to amplify 3∼6 CpG dinucleotides sites in each gene region, including four conserved regions among *ALVE1*, *ALVE2* and *ALVE3*, a unique region in *ALVE1* [48], and one promoter region of *TVB* gene (Figure 1 and Table 1). Forward and reverse primers used in PCR and the sequencing primers used in pyrosequencing methylation assays were designed with PSQ Assay Design software (Biotage, Swedan). To save time and cost, a biotin labeled universal primer (5′-GGGACACCGCTGATCGTTTA-3′) was used in the PCR assays [29]. The 5′ end of each reverse primer was tailed with the same sequence as the universal primer (Table 1).

**Table 1:** PCR and pyrosequencing primers and assays for each gene.

Gene^A^	Assay	CpG sites^B^	Primers	Sequence^C^
	6: 882	892	Forward	5′-TTAGGGGGAGGGAGGGTTT-3′
*ALVE*-region1	905	911	Reverse	5′-GGGACACCGCTGATCGTTTA CCATCTTCACATCTCGCTACACAA-3′
	914	919	Sequencing	5′-GAGGGTTTTTTTTTTAGGT-3′
			Assay	5′-T**Y**GGAGTGTA T**Y**GAGAAATT AGTAA**Y**GGAG **Y**GG**Y**GAAT**Y**G-3′
	5: 7171	7178	Forward	5′-GTATATGGGTGGTGGTATGAAATTTG-3′
*ALVE*-region2	7186	7194	Reverse	5′-GGGACACCGCTGATCGTTTA TTCCCCCTCCCTATACAAAAAC-3′
	7196		Sequencing	5′-GAGGGGATTATAGTATGTAT-3′
			Assay	5′-AGG**Y**GAAAGG **Y**GGGGTTT**Y**G GTTGTA**Y**G**Y**G GTTAGGAGTT-3′
	3′ LTR	5′LTR	Forward	5′-TGGYGATTAGATAAGGAAGGAATG-3′
*ALVE* -region3/4	4: 7359	4: 108	Reverse	5′-GGGACACCGCTGATCGTTTA TATCCATCTACCCAAATACACACCA-3′
	7375	124	Sequencing	5′-YGATTAGATAAGGAAGGAAT-3′
	7381	130	Assay	5′-GA**Y**GTAAGGA TATATGGG**Y**G TAGA**Y**GAAGT TATGTA**Y**GATTATATAAGTT-3′
	7393	142		
	4: 3793		Forward	5′-AGGCGTTTATTGTTTGGTTAGAAG-3′
*ALVE* -region5	3795		Reverse	5′-GGGACACCGCTGATCGTTTA CAAAAAAATATCAACCTCCTTACC-3′
	3800		Sequencing	5′-TTATTTTTTTGATTATTAAG-3′
	3808		Assay	5′-TTA**Y**G**Y**GTTT **Y**GGTAGTG**Y**G AATTTTTGGT AAGGAGGTTG ATAT-3′
			Forward	5′-ATGTGTAGGTTATGGGAAGGGTAT-3′
*TVB*	3: 1282407		Reverse	5′-GGGACACCGCTGATCGTTTA AAAACTAAACTACTCCCACCATTT-3′
	1282435		Sequencing	5′-GGTTATGGGAAGGGTA-3′
	1282444		Assay	5′-TAA**Y**GTGGTT ATGGTGTTTT TGTTTGGTTG T**Y**GTTGTTTA **Y**GTA-3′
			Universal	5′-/Biotin labeled/GGGACACCGCTGATCGTTTA-3′

A: *ALVE*-region1, 2, 3/4: based on the conserved DNA sequence between *ALVE1* (AY013303) and *ALVE3* (AY013304). The relative product of *ALVE* region1 is p19 matrix protein of *gag* gene. *ALVE* region2 is the consensus sequence of oncogene Direction Repeat Sequence (DRS). *ALVE* region3/4 is the conserved DNA sequence-U3 region, which located in 5′*LTR* and 3′*LTR* of *ALVE*s. *ALVE* region5: based on the unique DNA sequence (reverse transcriptase alpha subunit) in *ALVE1* (AY013303,[48]. *TVB*: based on the UCSC DNA sequence (May 2006, Chr22) that BLAT from *TVB* cDNA sequence (AF161712), it is on the promoter region of *TVB*.

B: The CpG site numbers and positions.

C: Y and R stand for C/T and G/A, respectively. Bold Y is the CpG sites assayed in each region.

### Hot start PCR amplification

The hot start PCR was carried out in 30 µl solution for ALVE and TVB genes: 1.5 µl bisulfite treated DNA (1∶5 dilution), 1×PCR buffer, 0.2 mM dNTPs, 0.5 µM forward primer, 0.05 µM reverse primer with universal tail, 0.45 µM biotin labeled universal primer, and 0.75 U Qiagen's Hotstar Taq DNA polymerase. PCR cycling conditions were 95°C for 15 min, followed by 50 cycles at 94°C for 30 sec, 50∼60°C for 45 sec, and 72°C for 45 sec, and a final incubation at 72°C for 10 min. PCR product quality verification was defined using 1.5% agarose gels with ethidium bromide.

### Pyrosequencing methylation analysis

Based on the concentration of the PCR product, 10∼25 µl PCR product was used for each pyrosequencing reaction. Pyrosequencing methylation analysis was carried out using the Pyro Q-CpG system (PyroMark ID, Biotage, Sweden) according to the manufacture's protocol. In brief, the PCR product was bound to Streptavidin coated Sepharose beads (GE Healthcare Bio-sciences AB, Sweden). The Sepharose beads containing the immobilized PCR product were purified in 70% ethanol for 5 sec, denatured in Denature buffer (Biotage) for 5 sec, and washed with Washing buffer (Biotage) for 10 sec using the pyrosequencing Vacuum Prep Tool (Biotage). Then, 0.5 µM sequence primer was annealed to the purified single-stranded PCR product and pyrosequencing was carried out using the Pyro Q-CpG system. The level of methylation was expressed for each cytosine locus on CpG sites as the percentage of ^m^C/(^m^C+C) (Figure 2, ^m^C is methylated cytosine, C is unmethylated cytosine). Non-CpG cytosine residues were used as controls to verify bisulfite conversion.

### Real-time RT-PCR

Total RNA of 5 individuals from each line was extracted from liver, spleen and hypothalamus using an RNeasy Midi kit (Qiagen). The first strand cDNA was synthesized from total RNA using SuperScript™ III Reverse Transcriptase (Invitrogen). Samples were then analyzed by real time RT-PCR using an iCycler iQ PCR system (Bio-Rad). The real time RT-PCR reactions were performed in a final volume of 20 µl with a QuantiTect SYBR Green PCR Kit (Qiagen) according to the manufacture's instructions. The mRNA expression of *PPT* (Polypurine tract) with *U3* region (termed as *PPT-U3*) on 3′ *LTR* of *ALVE* and *TVB* was normalized against the housekeeping gene *GAPDH* (glyceraldehyde-3-phosphate dehydrogenase) cDNA in the corresponding samples.

### Statistical analysis

Statistical analyses were conducted with the SAS 9.1.3 package. Point-wise comparison was carried out to analyze the difference of methylation contents between two lines at different CpG sites. Student's *t* test was done for analyzing mRNA expression levels of the *PPT-U3* region in 3′-*ALVE* between line 6_3_ and line 7_2_. The GLM (generalized linear model) program was used to analyze the association between the RNA expression levels and lines, tissues or DNA methylation levels. The first model is: *y* = *μ*+*l*+*t*+*e*, where *y* is the RNA expression level of *PPT-U3* region in 3′*LTR* of *ALVE*, *µ* is the overall mean, *l* is the class of line (Line 6_3_ and Line 7_2_), *t* is the type of tissue (liver, spleen or hypothalamus), and *e* is residual effect. The second model is: y = *μ*+m+t+e, where *y*, *µ* , *t* and *e* have the same meaning as that in the first model, and *m* is the DNA methylation level of *ALVE* region 2 or the promoter region in *TVB*, respectively. We also did the regression analysis of DNA methylation contents of *ALVE* region 1, 2 and 3/4 on mRNA expression level of the *PPT-U3* region in *ALVEs*.

## Supporting Information

Table S1 The methylation percentage (%) of ALVE-region1 in line 6_3_ and line 7_2_
(0.03 MB TIF)

Table S2 The methylation percentage (%) of ALVE-region2 in line 6_3_ and line 7_2_
(0.03 MB DOC)

Table S3 The methylation percentage (%) of ALVE-region3/4 in line 6_3_ and line 7_2_
(0.03 MB DOC)

Figure S1 PCR diagnostics for *ALVE1*, *ALVE2* and *ALVE3* in line 6_3_ and line 7_2_. *n* = 3 for each line. L63: line 6_3_; L72: line 7_2_. M: 100 bp markers. “-” is negative control. A. Left panel of Marker lane shows that line 6_3_ and line 7_2_ are all positive *ALVE1* birds. Right panel of Marker lane shows that line 7_2_ is *ALVE2* positive birds, however, line 6_3_ is *ALVE2* negative birds. B. Line 6_3_ is *ALVE3* positive birds, and line 7_2_ is *ALVE3* negative birds.
(0.99 MB DOC)

Figure S2 Validation of pyrosequencing results by bisulfite cloning and sequencing methods. TA Cloning Kit (Invitrogen Inc.) was used in cloning. The sequencing was done by ABI 3730. Black dots show methylated CpG sites, while open dots show unmethylated CpG sites. The same bisulfite treated spleen DNA from line 7_2_ (A) and line 6_3_ (B) was tested with cloning and sequencing (right panel) and pyrosequencing (left panel).
(2.12 MB TIF)

Figure S3 Regression analysis of mRNA expression level of *PPT-U3* region of *ALVE* and DNA methylation contents of *ALVE* region1 (Figure 3A) and *ALVE* region3/4 (Figure 3B).
(0.87 MB TIF)
