# Supplementary figures and images for: Quantitative Evaluation of DNA Methylation Patterns for ALVE and TVB Genes in a Neoplastic Disease Susceptible and Resistant Chicken Model
Source: PLoS One. 2008 Mar 5;3(3):e1731. doi: 10.1371/journal.pone.0001731 (PMC2254315; doi:10.1371/journal.pone.0001731)

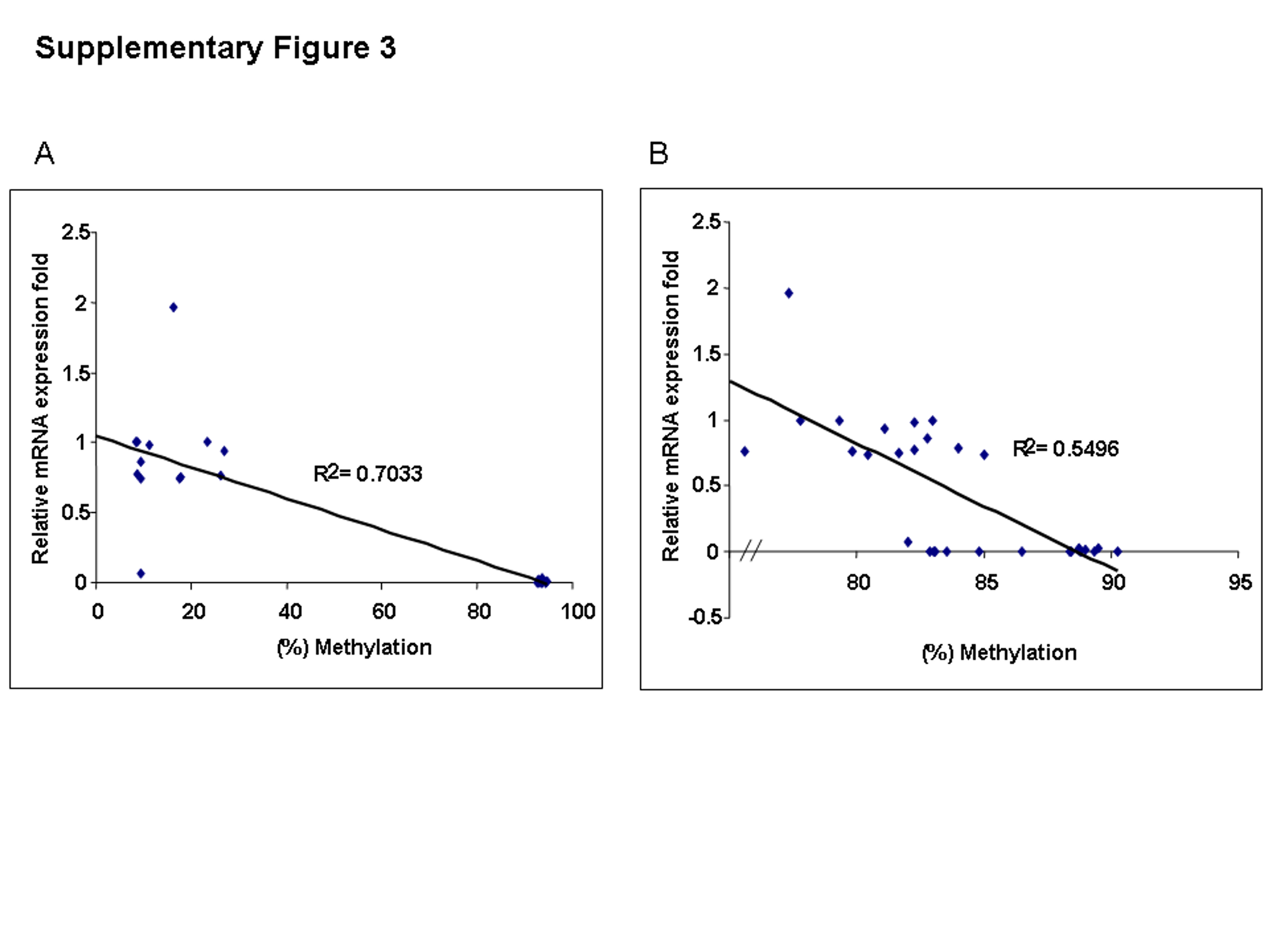

Supplement: Table S1 — The methylation percentage (%) of ALVE-region1 in line 63 and line 72 (0.03 MB TIF) [file pone.0001731.s001.tif]

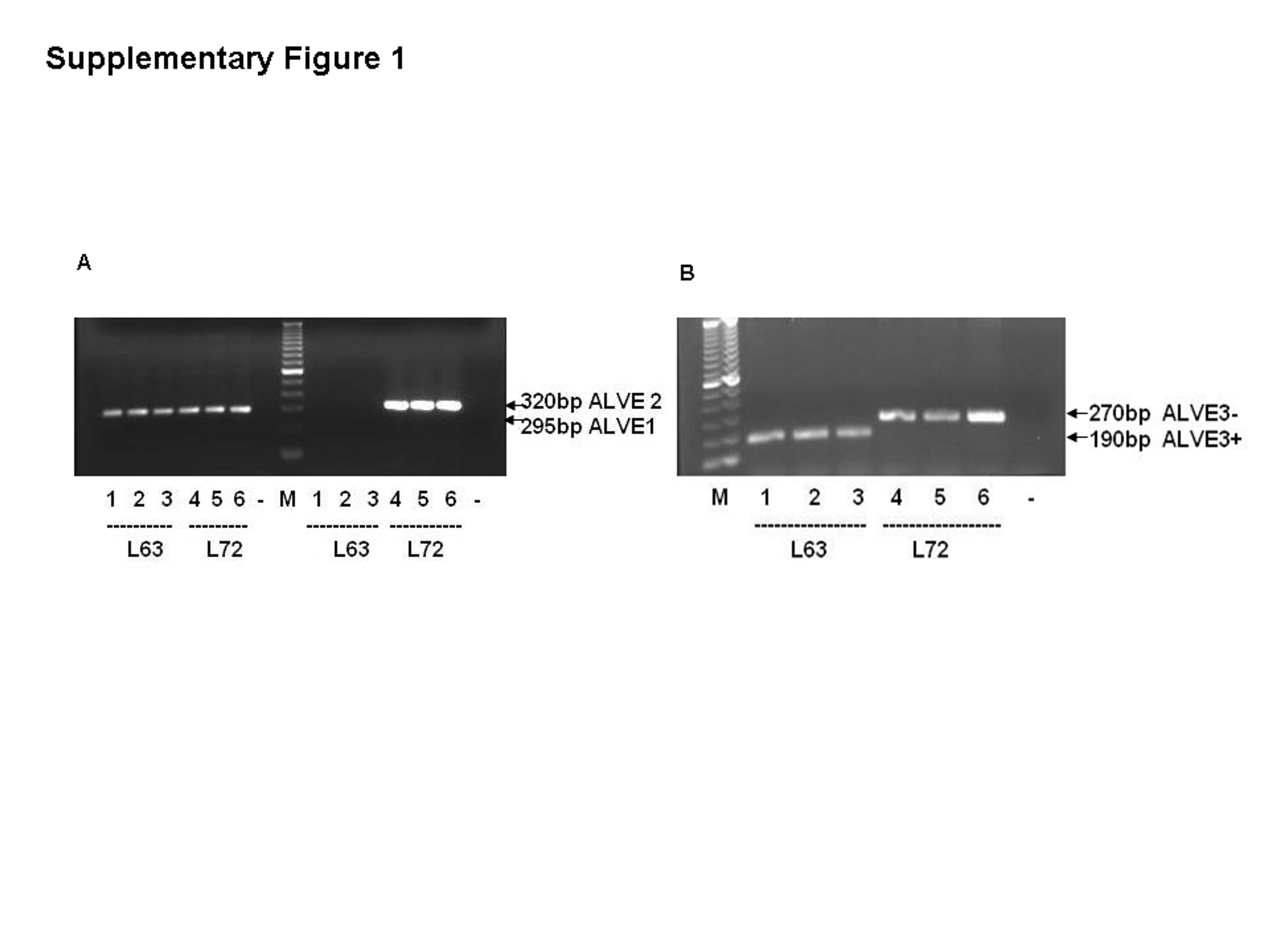

Supplement: Figure S2 — Validation of pyrosequencing results by bisulfite cloning and sequencing methods. TA Cloning Kit (Invitrogen Inc.) was used in cloning. The sequencing was done by ABI 3730. Black dots show methylated CpG sites, while open dots show unmethylated CpG sites. The same bisulfite treated spleen DNA from line 72 (A) and line 63 (B) was tested with cloning and sequencing (right panel) and pyrosequencing (left panel). (2.12 MB TIF) [file pone.0001731.s005.tif]

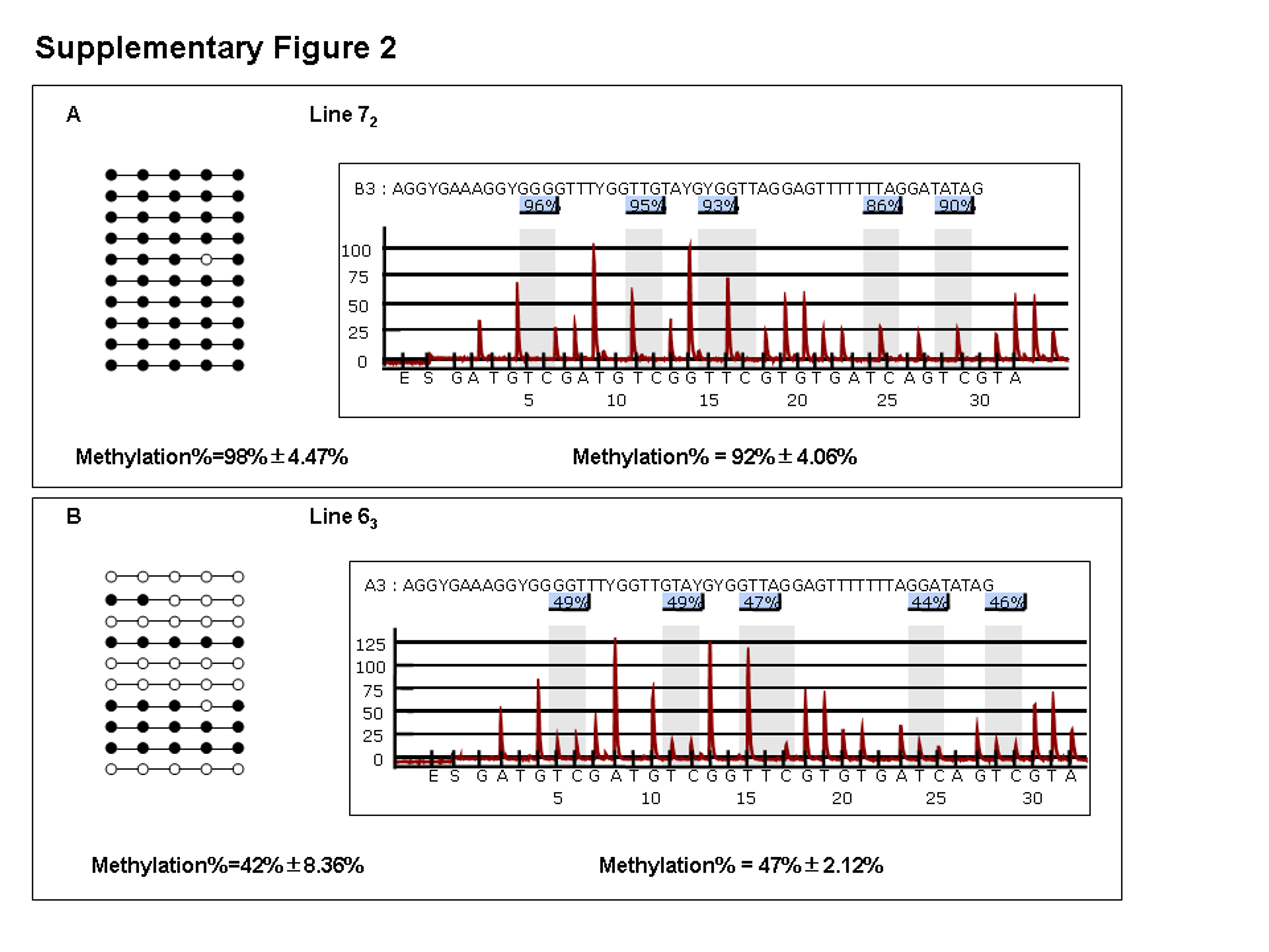

Supplement: Figure S3 — Regression analysis of mRNA expression level of PPT-U3 region of ALVE and DNA methylation contents of ALVE region1 (Figure 3A) and ALVE region3/4 (Figure 3B). (0.87 MB TIF) [file pone.0001731.s006.tif]
