# Supplementary material for: Quantitative Evaluation of DNA Methylation Patterns for ALVE and TVB Genes in a Neoplastic Disease Susceptible and Resistant Chicken Model
Source: PLoS One. 2008 Mar 5;3(3):e1731. doi: 10.1371/journal.pone.0001731 (PMC2254315; doi:10.1371/journal.pone.0001731)
Supplement: Table S2 — The methylation percentage (%) of ALVE-region2 in line 63 and line 72 (0.03 MB DOC) [file pone.0001731.s002.doc]

Table S1. The methylation percentage (%) of *ALVE*-region1 in line 6_3_ and line 7_2_

| CpG sites | L7_2_-Spleen | | L6_3_-Spleen | | L7_2_-Liver | | L6_3_-Liver | | L7_2_-Hypothalamus | | L6_3_-Hypothamalus | |
| --- | --- | --- | --- | --- | --- | --- | --- | --- | --- | --- | --- | --- |
|  | Mean^a^ | STD^b^ | Mean | STD | Mean | STD | Mean | STD | Mean | STD | Mean | STD |
| 1 | 98.44 | 1.85 | 13.89 | 4.72 | 99.45 | 1.07 | 15.79 | 7.09 | 95.87 | 2.95 | 18.94 | 10.85 |
| 2 | 99.22 | 1.30 | 12.40 | 4.82 | 97.98 | 1.72 | 14.84 | 6.64 | 95.18 | 3.13 | 18.84 | 12.60 |
| 3 | 89.14 | 2.53 | 9.67 | 3.10 | 85.73 | 4.39 | 12.23 | 4.76 | 90.72 | 1.17 | 14.10 | 6.70 |
| 4 | 88.29 | 4.91 | 12.20 | 4.26 | 95.45 | 4.25 | 17.80 | 10.17 | 92.67 | 1.83 | 17.13 | 9.65 |
| 5 | 86.40 | 1.36 | 11.18 | 4.00 | 84.76 | 3.45 | 13.83 | 6.99 | 88.03 | 2.11 | 17.61 | 10.43 |
| 6 | 99.31 | 1.00 | 14.19 | 5.90 | 97.70 | 3.22 | 17.09 | 5.41 | 99.49 | 1.02 | 19.45 | 11.46 |

^a^: Average value of methylation percentage for each CpG site, *n*=5 for each line and each tissue. ^b^: Standard deviation.
