# Supplementary material for: Quantitative Evaluation of DNA Methylation Patterns for ALVE and TVB Genes in a Neoplastic Disease Susceptible and Resistant Chicken Model
Source: PLoS One. 2008 Mar 5;3(3):e1731. doi: 10.1371/journal.pone.0001731 (PMC2254315; doi:10.1371/journal.pone.0001731)
Supplement: Table S3 — The methylation percentage (%) of ALVE-region3/4 in line 63 and line 72 (0.03 MB DOC) [file pone.0001731.s003.doc]

Table S2. The methylation percentage (%) of *ALVE*-region2 in line 6_3_ and line 7_2_

| CpG | L7_2_-Spleen | | L6_3_-Spleen | | L7_2_-Liver | | L6_3_-Liver | | L7_2_-Hypothalamus | | L6_3_-Hypothalamus | |
| --- | --- | --- | --- | --- | --- | --- | --- | --- | --- | --- | --- | --- |
| site | Mean^a^ | STD^b^ | Mean | STD | Mean | STD | Mean | STD | Mean | STD | Mean | STD |
| 1 | 95.01 | 2.11 | 48.13 | 1.59 | 93.67 | 2.31 | 57.89 | 4.91 | 92.72 | 9.22 | 77.67 | 1.94 |
| 2 | 93.54 | 1.84 | 47.61 | 2.65 | 93.62 | 1.38 | 56.95 | 5.19 | 91.81 | 8.31 | 76.05 | 2.47 |
| 3 | 93.08 | 1.68 | 46.10 | 2.68 | 90.68 | 5.30 | 54.25 | 5.06 | 85.34 | 11.88 | 66.64 | 1.33 |
| 4 | 88.38 | 2.13 | 43.39 | 3.00 | 85.27 | 3.38 | 47.20 | 9.58 | 69.21 | 12.00 | 44.49 | 1.34 |
| 5 | 89.31 | 2.99 | 45.59 | 3.23 | 89.35 | 4.18 | 51.96 | 5.59 | 79.48 | 12.15 | 54.72 | 3.26 |

^a^: Average value of methylation percentage for each CpG site, *n*=5 for each line and each tissue. ^b^: Standard deviation.
