# Supplementary material for: Quantitative Evaluation of DNA Methylation Patterns for ALVE and TVB Genes in a Neoplastic Disease Susceptible and Resistant Chicken Model
Source: PLoS One. 2008 Mar 5;3(3):e1731. doi: 10.1371/journal.pone.0001731 (PMC2254315; doi:10.1371/journal.pone.0001731)
Supplement: Figure S1 — PCR diagnostics for ALVE1, ALVE2 and ALVE3 in line 63 and line 72. n = 3 for each line. L63: line 63; L72: line 72. M: 100 bp markers. “-” is negative control. A. Left panel of Marker lane shows that line 63 and line 72 are all positive ALVE1 birds. Right panel of Marker lane shows that line 72 is ALVE2 positive birds, however, line 63 is ALVE2 negative birds. B. Line 63 is ALVE3 positive birds, and line 72 is ALVE3 negative birds. (0.99 MB DOC) [file pone.0001731.s004.doc]

Table S3. The methylation percentage (%) of *ALVE*-region3/4 in line 6_3_ and line 7_2_

| CpG sites | L7_2_-Spleen | | L6_3_-Spleen | | L7_2_-Liver | | L6_3_-Liver | | L7_2_-Hypothalamus | | L6_3_-Hypothalamus | |
| --- | --- | --- | --- | --- | --- | --- | --- | --- | --- | --- | --- | --- |
|  | Mean^a^ | STD^b^ | Mean | STD | Mean | STD | Mean | STD | Mean | STD | Mean | STD |
| 1 | 94.85 | 0.87 | 85.57 | 3.01 | 92.31 | 1.03 | 90.05 | 2.69 | 95.52 | 1.62 | 88.67 | 3.86 |
| 2 | 91.81 | 0.68 | 84.22 | 2.22 | 89.53 | 1.25 | 86.40 | 3.03 | 92.20 | 1.01 | 86.07 | 3.09 |
| 3 | 84.91 | 1.17 | 75.51 | 2.89 | 77.15 | 1.68 | 74.05 | 3.95 | 82.51 | 1.25 | 74.13 | 2.74 |
| 4 | 84.69 | 0.62 | 74.96 | 3.06 | 74.99 | 1.41 | 76.48 | 1.68 | 83.69 | 1.84 | 75.44 | 3.47 |

^a^: Average value of methylation percentage for each CpG site, *n*=5 for each line and each tissue. ^b^: Standard deviation.
